# Lipids, lipid-lowering agents, and inflammatory bowel disease: a Mendelian randomization study

**DOI:** 10.3389/fimmu.2023.1160312

**Published:** 2023-06-07

**Authors:** Heqing Tao, Zhou Yu, Yongqiang Dong, Ligang Liu, Liang Peng, Xueqing Chen

**Affiliations:** ^1^ Department of Gastroenterology, The First Affiliated Hospital of Guangzhou Medical University, Guangzhou Medical University, Guangzhou, China; ^2^ Department of Neurology, Peking University Third Hospital, Beijing, China; ^3^ Deartment of Thyroid Surgery, First Affiliated Hospital of Zhengzhou University, Zhengzhou, China; ^4^ Institute of Therapeutic Innovations and Outcomes, College of Pharmacy, The Ohio State University, Columbus, OH, United States

**Keywords:** lipids, lipid-lowering, inflammatory bowel disease, Mendelian randomization, *PCSK9*, *CTEP 2*

## Abstract

**Background:**

To assess the causal role of lipid traits and lipid-lowering agents in inflammatory bowel disease (IBD).

**Methods:**

Univariable mendelian randomization (MR) and multivariable MR (MVMR) analyses were conducted to evaluate the causal association between low-density lipoprotein cholesterol (LDL-C), triglycerides (TG), high-density lipoprotein cholesterol (HDL-C) and IBD. Drug-targeted MR analyzed the effects of lipid-lowering drugs on IBD, and network MR was used to analyze potential mediation effects.

**Results:**

The levels of HDL-C had an inverse relationship with the risk of Crohn’s disease (CD, OR: 0.85, 95% CI: 0.73-0.98, *P* = 0.024). In MVMR, the inverse relationships were found in all three outcomes. Drug-targeted MR analyses showed that with one-SD LDL-C decrease predicted by variants at or near proprotein convertase subtilisin/kexin type 9 (*PCSK9)*, the OR values of people diagnosed with IBD, ulcerative colitis (UC) and CD were 1.75 (95%CI: 1.13-2.69, *P* = 0.011), 2.1 (95%CI: 1.28-3.42, *P* = 0.003) and 2.24 (95%CI: 1.11-4.5, *P* = 0.024), respectively. With one-SD LDL-C decrease predicted by variants at or near cholesteryl ester transfer protein *(CETP)*, the OR value of people diagnosed with CD was 0.12 (95%CI: 0.03-0.51, *P* = 0.004). Network-MR showed that HDL-C mediated the causal pathway from variants at or near *CETP* to CD.

**Conclusion:**

Our study suggested a causal association between HDL-C and IBD, UC and CD. Genetically proxied inhibition of *PCSK9* increased the risk of IBD, UC and CD, while inhibition of *CETP* decreased the risk of CD. Further studies are needed to clarify the long-term effect of lipid-lowering drugs on the gastrointestinal disorders.

## 1 Introduction

Inflammatory bowel disease (IBD) is chronic intestinal inflammation disease with an incidence of over 0.3%. It includes ulcerative colitis (UC) and Crohn’s disease (CD), causing a significant disease burden (1). Patients with IBD often have high levels of low-density lipoprotein cholesterol (LDL-C), triglycerides (TG), and low level of high-density lipoprotein cholesterol (HDL-C) (2, (3). Dyslipidemia may also contribute to the development of IBD. Koutroumpakis et al. found that persistent dyslipidemia was associated with disease activity in patients with IBD (3). Lower HDL-C was associated with an increased risk of poor outcomes, such as surgery and tumors in IBD patients (4). Additionally, evidence suggests that lower HDL-C in childhood may be associated with the subsequent diagnosis of IBD (5).

In recent years, some researchers have recommended early evaluation and intervention of lipid levels to prevent new-onset IBD and improve the prognosis in patients with IBD. Statins, a widely used medication to lower LDL-C, have been found to have a protective effect against new-onset IBD, CD, and UC (6). Similarly, a population-based case-control study in Sweden showed that statins were associated with a lower risk of CD (7). In addition, the use of statins in UC patients was found to be associated with a reduction in steroid hormone dosage (8). Furthermore, transcriptomic analysis found that atorvastatin had the highest negative correlation with the UC gene signature, suggesting that statins may be potential therapies for IBD (9). However, the therapeutic effect of statins on IBD is controversial (10). Khalil et al. found that statins did not prevent new-onset IBD (11). Dhamija et al. reported that statin therapy was not associated with beneficial effects in patients with UC (10).

The contradictory findings in these studies highlight the need for further investigation into the potential role of statins in IBD. However, observational studies are limited by inherent constraints, including residual confounding and reverse causality (12). Additionally, lipid-lowering drugs have extensive immunomodulatory effects (13). which raises the question of whether their effect on IBD is mediated through lipid lowering or immunomodulatory mechanisms. Therefore, it is essential to elucidate the underlying mechanisms of the potential therapeutic effect of statins in IBD.

Mendelian randomization (MR) is a powerful tool for assessing the causal effects of exposure factors on outcomes. It utilizes genetic variation randomly allocated at conception as instrumental variables (IVs), allowing for the evaluation of causality in a way that is less susceptible to confounding factors (12). Univariate MR requires that the instrumental variable satisfies three assumptions: first, it must be associated with the exposure; second, it must be independent of the outcome given the exposure; and third, it must be independent of all known confounders (14). Multivariate MR (MVMR) is an extension of MR that leverages genetic variation associated with multiple potentially relevant exposures to estimate the direct effect of each exposure on a single outcome (14). Drug-target MR analysis uses the genetic variants within or near the gene that encodes the targeted protein as IVs to predict efficacy (15). In this study, we applied the latest genome-wide association study (GWAS) from UK Biobank on lipids as IVs to study the relationship between lipids and IBD. Additionally, we employed drug-target MR analysis to investigate the effect of lipid-lowering drugs on IBD.

## 2 Materials and methods

### 2.1 GWAS data source

The data used in this study on IBD patients were obtained from the FinnGen study conducted in 2021 (16). The study included patients with UC, CD, and indeterminate colitis, with UC and CD diagnosed by ICD codes. Information of participants, genotype platforms are available at the FinnGen website [https://www.finngen.fi/en/]. The dataset used comprised of 5,673 IBD patients and 213,119 control patients, including 4,320 patients with UC and 210,300 controls, as well as 2,056 patients with CD and 210,300 controls To minimize bias resulting from racial differences, the SNPs identified as instrumental variables (IVs) associated with lipid traits were obtained from the UK Biobank (17), and only SNPs from studies based on European ancestry were selected.

### 2.2 Instrumental variable selection

In the univariable MR analyses, we applied rigorous quality control procedures to identify independent, eligible, and genome-wide significant SNPs (linkage disequilibrium, LD clumping r2 threshold = 0.001, window size = 10 Mb and *p* < 5×10^-8^) associated with each trait, including HDL-C, LDL-C and TG. For MVMR analyses, SNPs were clumped with respect to the lowest *P*-value corresponding to each exposure in a multivariable model using a 1-Mb window and pairwise LD R^2^ < 0.001. F-statistic was used to access the strength of instruments in univariable MR, and conditional F-statistic to access the strength in MVMR. The value of a statistic less than 10 were excluded to reduce weak instrument bias. We obtained the lipid-lowering drug targets related IVs from the Global Lipid Genetics Consortium (GLGC) for Drug-target MR analyses.

The IVs for lipid-lowering drug targets were from a GWAS of LDL-C conducted by the Global Lipid Genetics Consortium (GLGC) (linkage disequilibrium, LD r2 ≤ 0.2, physical distance = 250 kb, window size =100 kb and *p* < 5×10^-8^) (18). The targets included 3-Hydroxy-3-Methylglutaryl-CoA Reductase (*HMGCR*), NPC1 Like Intracellular Cholesterol Transporter 1(*NPC1L1*), Proprotein convertase subtilisin/kexin type 9 (*PCSK9*), Apolipoprotein B (*APOB*), Cholesteryl ester transfer protein (*CETP*), with each gene including 5,4,11,15,7 SNP that were used as IVs. Each lipid-lowering drug target corresponds to statins, ezetimibe, evolocumab & alirocumab, anacetrapib and mipomersen, separately. For each SNP, the effect allele is significantly associated with lower concentrations of LDL-C. We ensured that there were no strong correlations between IVs of each trait (R2 < 0.4 or R2 < 0.3).

### 2.3 MR analyses

**Figure 1:**
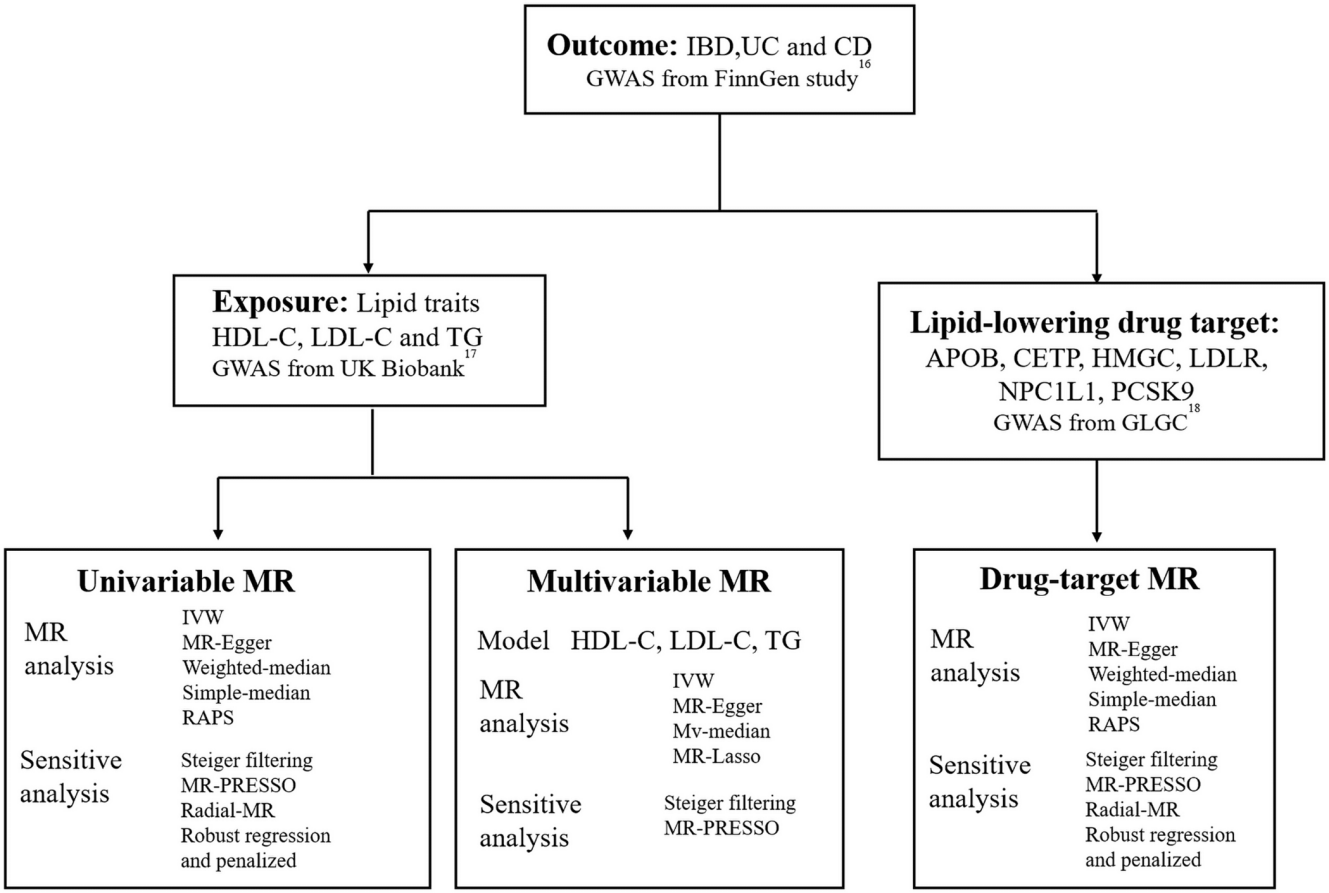
Overview of the study design. GWAS, genome-wide association study; IBD, inflammatory bowel disease; UC, ulcerative colitis; CD, Crohn’s disease; HDL-C, high-density lipoprotein cholesterol; LDL-C, low-density lipoprotein cholesterol; TG, triglycerides; IVW, inverse-variance weighted; RAPS, MR robust adjusted profile score; HMGCR, 3-Hydroxy-3-Methylglutaryl-CoA Reductase; NPC1L1, NPC1 Like Intracellular Cholesterol Transporter 1; PCSK9, Proprotein convertase subtilisin/kexin type 9; APOB, Apolipoprotein B; CETP, Cholesteryl ester transfer protein; GLGC, Global Lipid Genetics Consortium; MR, Mendelian randomization.

**Figure 1** illustrates the different methods we employed to conduct univariable and multivariable Mendelian randomization (MR) analyses, including simple median, weighted median, MR-Egger, MR robust adjusted profile score (MR.RAPS) (19) and inverse-variance weighted (IVW) method. Fixed-effect model IVW, which offers high efficiency and statistical power, was the primary method used (20). To assess heterogeneity and pleiotropy, we used Cochran’s Q statistic and the MR-Egger test (intercept). In cases where significant heterogeneity was present, we utilized the multiplicative random effects IVW method. Additionally, we employed the MR-radial method, robust regression, and outlying variants penalized to investigate the impact of outliers on the outcome (21, (22). In instances where horizontal pleiotropy was detected, we employed the MR-Egger test as the primary analysis method, while using MR-PRESSO to further correct for pleiotropy (23). For multivariable MR (MVMR) analyses, we utilized the multivariable IVW, multivariable MR-Egger, multivariable median-based, and multivariable MR-Lasso methods. We used the multivariable MR-Egger method for pleiotropy testing and Cochran’s Q statistic for heterogeneity testing. All MR analyses adhered to the STROBE-MR Statement (24).

To investigate the causality of lipid-lowering drug targets in IBD,UC and CD, as well as the potential mediators involved, we performed network MR by conducting at least three two-sample MR tests among lipid-lowering drug targets, IBD/UC/CD, and potential mediators (25).

We used publicly available genome-wide association study (GWAS) data. Relevant informed consent and ethical approval had already been obtained, so additional ethical approval was not required for this study. We conducted all statistical analyses using the “TwoSample MR” (version 0.5.6) and “MendelianRandomization” (version 0.5.1) packages in R software (version 4.1.1), with a statistical significance threshold of P value < 0.05.

## 3 Results

We identified 525, 218, and 435 SNPs as instrumental variables (IVs) for HDL-C, LDL-C, and TG, respectively (**Supplementary Table 1**). The median F statistic of these IVs was 47.49 (with a quartile range of 35.56-84.49), indicating that our data were not susceptible to weak instrument bias.

### 3.1 Causal effect of lipids on IBD, UC and CD

Significant heterogeneity was observed between the independent variables (IVs) and the outcomes of IBD, UC, and CD, as indicated by Cochran’s Q test (*P* < 0.05) (**Supplementary Table 2**, **Supplemental Figures 1–3**).

**Figure 2:**
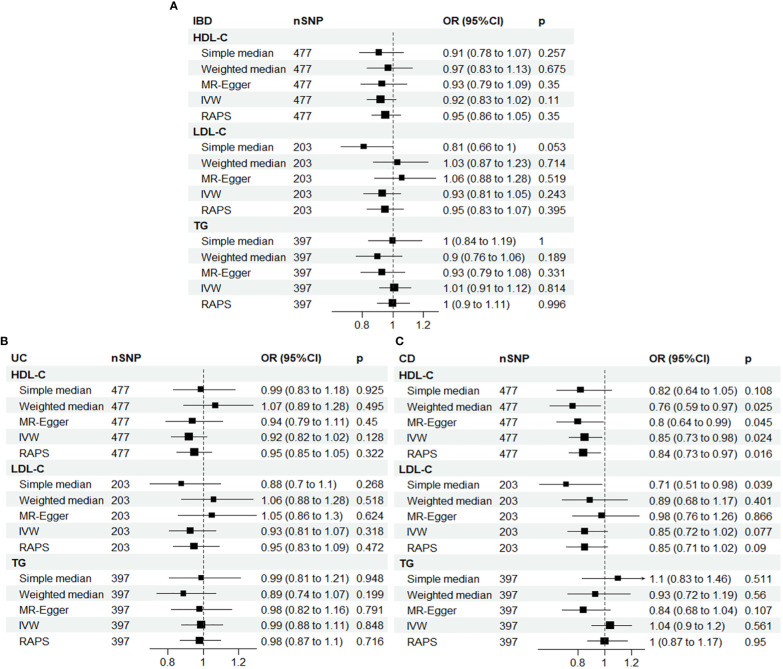
Univariable Mendelian randomization results using different methods. **(A)** IBD; **(B)** UC; **(C)** CD. IBD, inflammatory bowel disease; UC, ulcerative colitis; CD, Crohn’s disease; SNP N, number of single nucleotide polymorphisms; OR, odds ratio; CI, confidence interval; HDL-C, high-density lipoprotein cholesterol; LDL-C, low-density lipoprotein cholesterol; TG, triglycerides; IVW, inverse-variance weighted; RAPS, MR robust adjusted profile score.

Therefore, the multiplicative random effects model, known as the inverse variance weighted (IVW) method, was used as the main model. Univariate MR analyses revealed a significant association between HDL-C and CD, with an OR of 0.85 (95% CI: 0.73-0.98, *P* = 0.024) for a one-standard deviation increase in HDL-C, as shown in **Figure 2**.

To ensure the robustness of our results, we applied MR-radial method and robust regression and outlying variants (MR-RAPS) penalized analyses, which also showed a significant inverse association between HDL-C and CD, with ORs of 0.84 (95% CI: 0.73-0.97, *P* = 0.019) and 0.83 (95% CI: 0.72-0.96, *P* = 0.012), respectively, for a one-standard deviation increase in HDL-C. These findings are presented in **Supplementary Tables 3, 4** and **Supplemental Figures 4–7**.

Although some horizontal pleiotropy was detected in the MR analyses of HDL-C and IBD, and TG and CD, as shown by the MR-Egger test, the results of this test and the IVW analysis were consistent, indicating minimal impact of pleiotropy on the results. Furthermore, further analysis using the MR-PRESSO package yielded similar results, confirming the robustness of our findings (**Supplementary Table 5**).

### 3.2 Direct causal effect of lipids on IBD, UC and CD using multivariable MR

**Figure 3:**
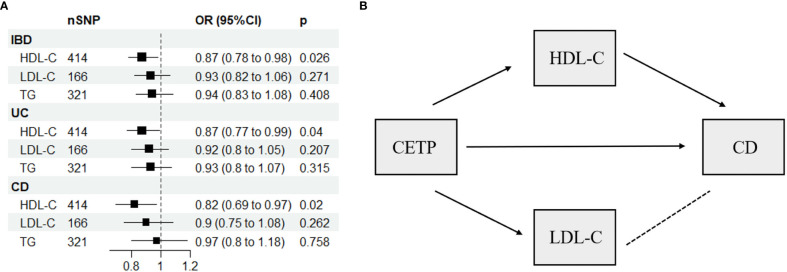
**(A)** Multivariable Mendelian randomization results using the inverse-variance weighted method. **(B)** Network-MR diagram of CETP and CD. IBD, inflammatory bowel disease; UC, ulcerative colitis; CD, Crohn’s disease; SNP N, number of single nucleotide polymorphisms; OR, odds ratio; CI, confidence interval; HDL-C, high-density lipoprotein cholesterol; LDL-C, low-density lipoprotein cholesterol; TG, triglycerides; CETP, Cholesteryl ester transfer protein, →, causal association; – – – –, no causal association.

The present study used a multivariable Mendelian randomization (MVMR) approach, which incorporated three variables (HDL-C, LDL-C, and TG) to evaluate potential weak instrumental variable bias. The conditional F-statistic values corresponding to these variables were 68.7, 35.46, and 62.2, respectively, indicating no significant bias. Our MVMR analyses further revealed that higher HDL-C levels were inversely associated with the risk of IBD (OR: 0.87, 95% CI: 0.78-0.98, *P* = 0.026), UC (OR: 0.87, 95% CI: 0.77-0.99, *P* = 0.04), and CD (OR: 0.82, 95% CI: 0.69-0.97, *P* = 0.02), as shown in **Figure 3A** and **Supplementary Table 6**. Notably, pleiotropy test did not detect significant pleiotropy, as summarized in **Supplementary Table 7**.

### 3.3 Gene-specific analyses for drug proxy variants on IBD, UC and CD

**Figure 4:**
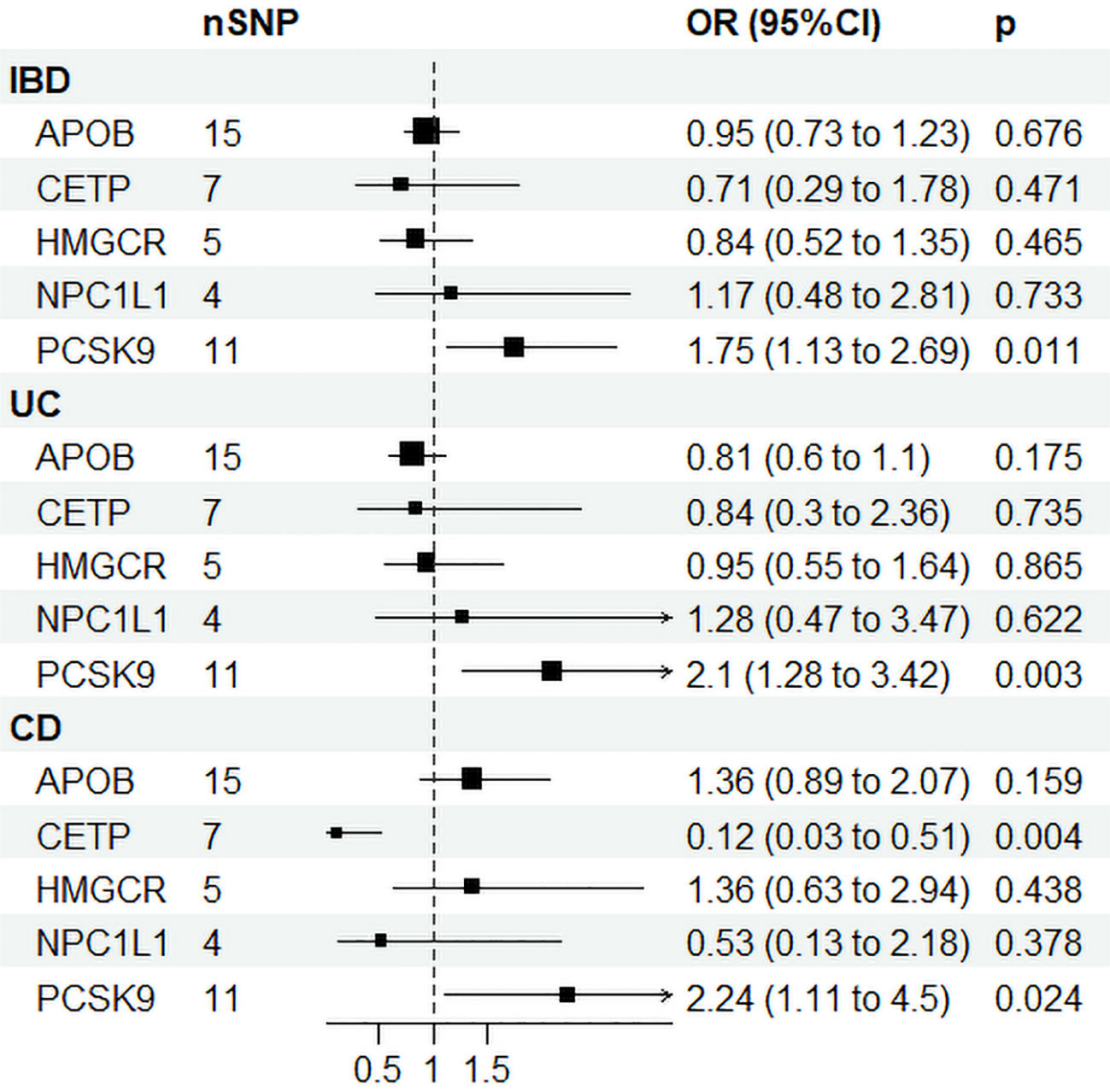
Drug target Mendelian randomization results. IBD, inflammatory bowel disease; UC, ulcerative colitis; CD, Crohn’s disease; SNP N, number of single nucleotide polymorphisms; OR, odds ratio; CI, confidence interval; HMGCR, 3-Hydroxy-3-Methylglutaryl-CoA Reductase; NPC1L1, NPC1 Like Intracellular Cholesterol Transporter 1; PCSK9, Proprotein convertase subtilisin/kexin type 9; APOB, Apolipoprotein B; CETP, Cholesteryl ester transfer protein.

**Supplementary Table 8** displays the IVs related to lipid-lowering drug targets. In **Figure 4**, drug-targeted MR analyses revealed that a one-SD decrease in LDL-C predicted by variants at or near *PCSK9* was associated with higher ORs of being diagnosed with IBD, UC, and CD with values of 1.75 (95%CI: 1.13-2.69, *P* = 0.011), 2.1 (95%CI: 1.28-3.42, *P* = 0.003), and 2.24 (95%CI: 1.11-4.5, *P* = 0.024), respectively. Conversely, a one-SD decrease in LDL-C predicted by variants at or near *CETP* was associated with a lower OR of CD with a value of 0.12 (95%CI: 0.03-0.51, *P* = 0.004), while no such association was observed for IBD or UC patients. **Figure 3B** and **Supplementary Table 9** demonstrated that HDL-C mediated the causal pathway from variants at or near *CETP* to CD in network-MR. Additionally, the heterogeneity analysis and pleiotropy test revealed no significant heterogeneity or pleiotropy (**Supplementary Table 10**). Furthermore, the leave-one-out analysis demonstrated that the drug-targeted MR results were robust, even after excluding a single single-nucleotide polymorphism (**Supplemental Figure 8**).

## 4 Discussion

To the best of our knowledge, this study is the first to utilize MR analysis to investigate the relationship between lipids, lipid-lowering drugs, and IBD. Our findings suggest a causal association between elevated levels of HDL-C and a reduced risk of all three IBD diseases, including UC and CD. Additionally, the drug-targeted MR analysis indicates that inhibiting *PCSK9* leads to an increased risk of all three diseases, while suppressing *CETP* reduces the risk of CD.

Our findings are consistent with previous studies that observed significant reductions in HDL-C levels in IBD patients (3), UC patients (26) and CD patients (27), indicating that HDL-C may play a role in the development of IBD. However, large prospective studies that evaluate the causal relationship between HDL-C and IBD are lacking. HDL-C is widely recognized as a complex and pleiotropic anti-inflammatory particle that plays a critical role in protecting the cardiovascular system by promoting cholesterol efflux in vascular endothelial cells, stimulating prostacyclin synthesis, and inhibiting platelet-activating factor synthesis (28). Whether HDL-C plays a protective role in the pathogenesis of IBD by inhibiting inflammation requires further investigation.

*CETP* primarily binds to HDL in the circulatory system, transporting cholesterol esters from HDL to LDL and VLDL (29). Our study found that inhibition of *CETP* could reduce the risk of CD. However, since variants in or near *CETP* are related to both HDL-C and LDL-C levels, it is challenging to differentiate whether the protective effect is due to changes in HDL-C or LDL-C. To address this issue, we conducted a network-MR analysis that indicated HDL-C, rather than LDL-C, mediated the causal relationship between *CETP* and CD. We did not find evidence of a mediating effect of *CETP* inhibitors on HDL-C in IBD and UC. This may be related to the complex metabolic pathways of HDL-C, which involve multiple components, including ATP-binding cassette transporter G1, ATP-binding cassette transporter A1, and lecithin cholesterol acyltransferase, in addition to *CETP* (30). Our study at least confirms that *CETP* does not regulate HDL-C in IBD and UC, suggesting the involvement of other molecules in its regulation that require further investigation.

In a previous MR study, no causal relationship was found between *CETP* and *PCSK9* inhibitors and IBD, UC, and CD (31). The discrepancy in results may be due to the different IVs used in our study, with our study using SNPs associated with LDL-C levels. Our study found a causal relationship between *PCSK9* inhibitors and IBD, UC, and CD. Interestingly, our study did not find a causal relationship between LDL-C and the three diseases, indicating that PCSK9 affects IBD, UC, and CD through means other than by reducing LDL-C. Although *PCSK9* was initially linked to elevated LDL-C, recent studies have found its involvement in atherosclerosis, viral infections, immune activation, and tumor progression (32, 33). While *PCSK9* is less expressed in the gastrointestinal tract, serum *PCSK9* levels were significantly higher in UC patients and associated with disease activity (34). The *PCSK9* inhibitor could inhibit the activity of TLR4/NF-κB to ameliorate colitis induced by 2,4,6-trinitrobenzene sulfonic acid (35). These findings appear to contradict our study, but further research is needed to fully understand the role of PCSK9 in the gastrointestinal tract. Studies using PCSK9 knockout mice have provided insight into interpreting our results. For instance, *PCSK9^-/-^* mice had more severe fibrosing steatohepatitis and were more prone to hepatocellular carcinoma (36). More severe oxidative stress was found in *PCSK99^-/-^* mice (37). In these studies, the variants of *PCSK9* variants involve a lifelong process that starts with embryonic development, which is different from the short-term effect of clinical drug use. Therefore, future research is necessary to explore the role of *PCSK9* in the gastrointestinal tract fully. Finally, our study did not establish a direct causal relationship between the *HMGCR* inhibitor and the three outcomes, suggesting that there is no direct causality between statins and IBD, UC, and CD.

Our study offers several notable strengths. Firstly, we utilized the largest lipid-related GWAS database to date, providing a robust theoretical basis for future treatment of IBD, UC, and CD by demonstrating the causal effect of HDL-C. Secondly, we employed drug target MR and network MR analysis to demonstrate the mediation effect of HDL-C in *CETP* on CD, and the causal effect of the *PCSK9* inhibitor on IBD, UC, and CD. These findings highlight the need for further research into the potential gastrointestinal side effects of long-term use of *PCSK9* inhibitors. Finally, we conducted detailed heterogeneity and pleiotropy tests, ensuring the reliability of our results. However, our study also has some limitations. The FinnGen study included only individuals of European ancestry, limiting the generalizability of our conclusions to other populations. Additionally, our drug target MR analysis reflects the impact of lifelong application of lipid-lowering drugs on outcomes, which may not accurately reflect the relationship between short-term use of these drugs and related outcomes.

## 5 Conclusions

In conclusion, this study provided strong evidence for the causal impact of HDL-C on IBD, UC, and CD. Furthermore, we have identified HDL-C as a mediator in the causal pathway linking CETP inhibitors to CD. Genetically proxied inhibition of *PCSK9* increased the risk of IBD, UC and CD, while inhibition of *CETP* decreased the risk of CD. However, further research is needed to investigate the potential role of PCSK9 inhibitors in gastrointestinal disorders.

## Data availability statement

The original contributions presented in the study are included in the article/**Supplementary Material**. Further inquiries can be directed to the corresponding authors.

## Ethics statement

The studies involving human participants were reviewed and approved by The ethics committee of The First Affiliated Hospital of Guangzhou Medical University. Written informed consent for participation was not required for this study in accordance with the national legislation and the institutional requirements.

## Author contributions

HT, ZY and YD planned and designed the study, analyzed and interpreted the data, and wrote the manuscript. LL and XC analyzed the data and made the tables presented in the manuscript. LP and YD, as corresponding authors, made important advice in the study design, supervised and coordinated the study conduct process, revised the manuscript and tables, as well as reviewed and verified all the data, methods, and results. All authors contributed to the article and approved the submitted version.
