# Supplementary material for: Lipids, lipid-lowering agents, and inflammatory bowel disease: a Mendelian randomization study
Source: Front Immunol. 2023 Jun 7;14:1160312. doi: 10.3389/fimmu.2023.1160312 (PMC10282130; doi:10.3389/fimmu.2023.1160312)
Supplement: Supplementary file 1 [file DataSheet_1.docx]

Supplementary Material

Lipids, Lipid-lowering agents, and inflammatory bowel disease: A Mendelian Randomization Study

Heqing Tao^1^†, Zhou Yu^2^†, Yongqiang Dong^3^†, Ligang Liu^4^, Liang Peng^1^ and Xueqing Chen^1^

*** Correspondence:** Liang Peng: wsfirefly@126.com

# Supplementary Figures and Tables

## Supplementary Figures

Supplemental Fig. 1. Scatter plots showing the effect of HDL-C on the outcome via different methods

Supplemental Fig. 1A represent the effect of the trait on all IBD


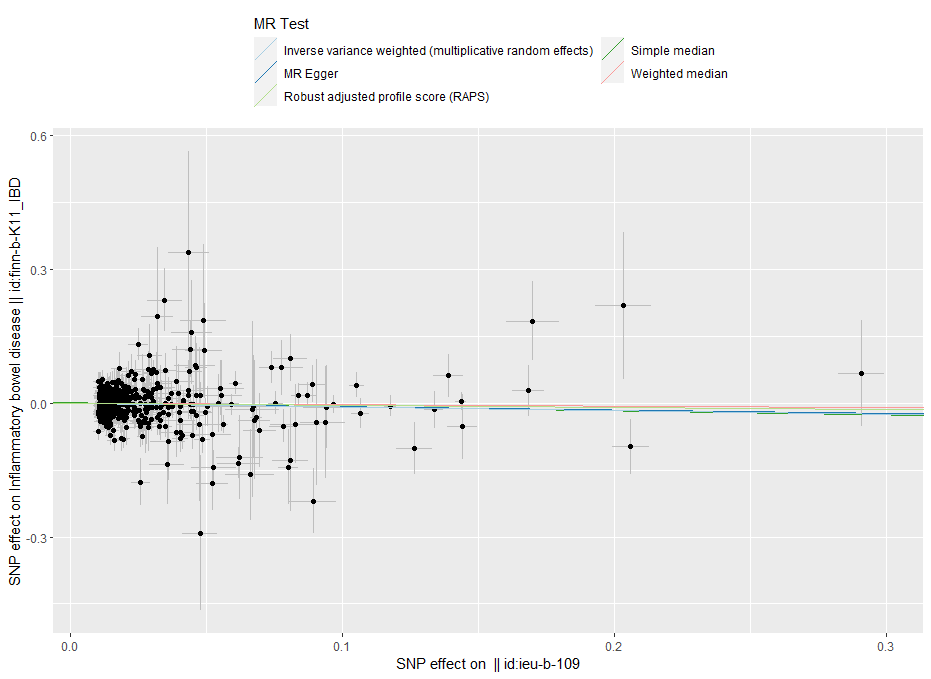


Supplemental Fig. 1B represent the effect of the trait on UC


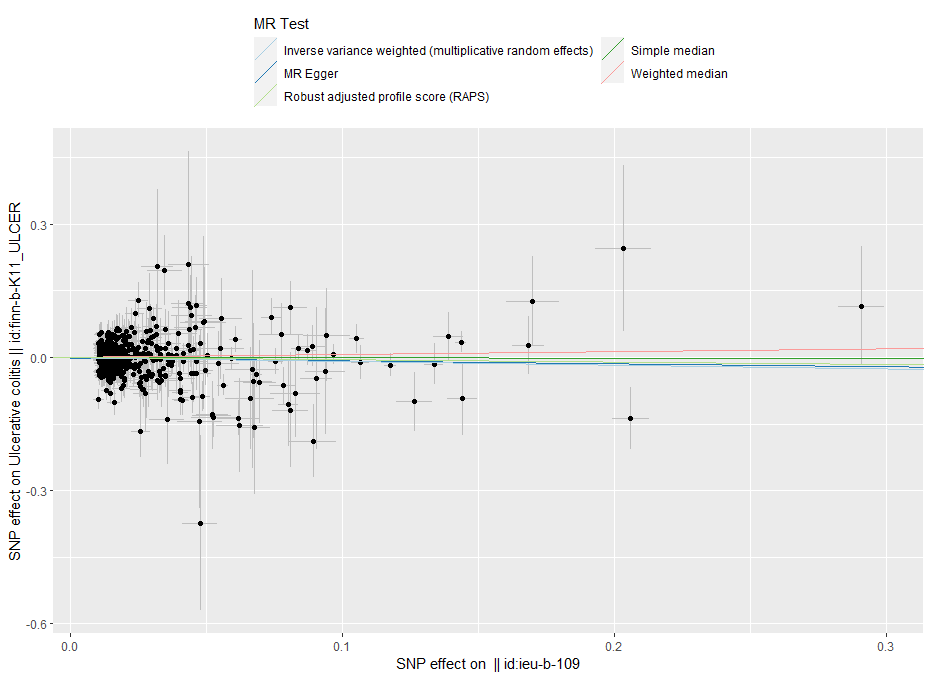


Supplemental Fig. 1C represent the effect of the trait on CD


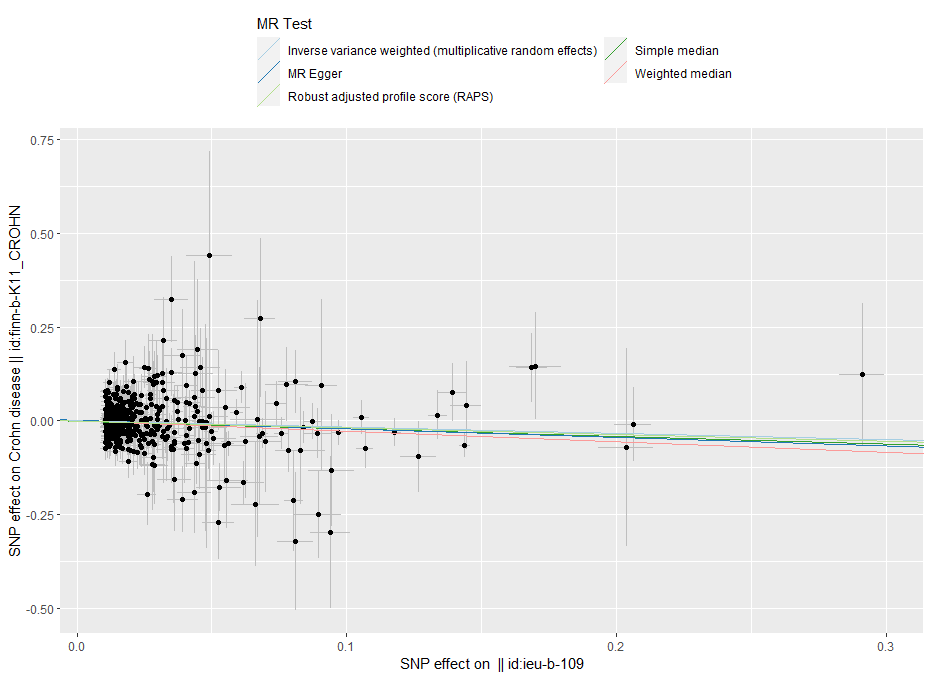


Supplemental Fig. 2. Scatter plots showing the effect of LDL-C on the outcome via different methods

(A), (B), (C) represent the effect of the trait on all IBD, UC and CD, respectively.

Supplemental Fig. 2A represent the effect of the trait on all IBD


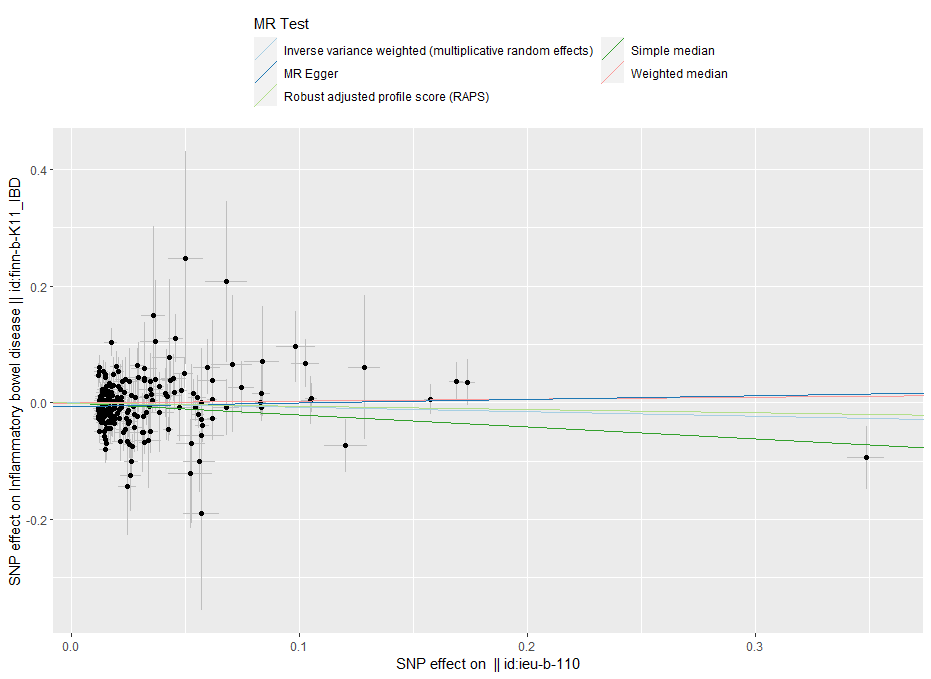


Supplemental Fig. 2B represent the effect of the trait on UC


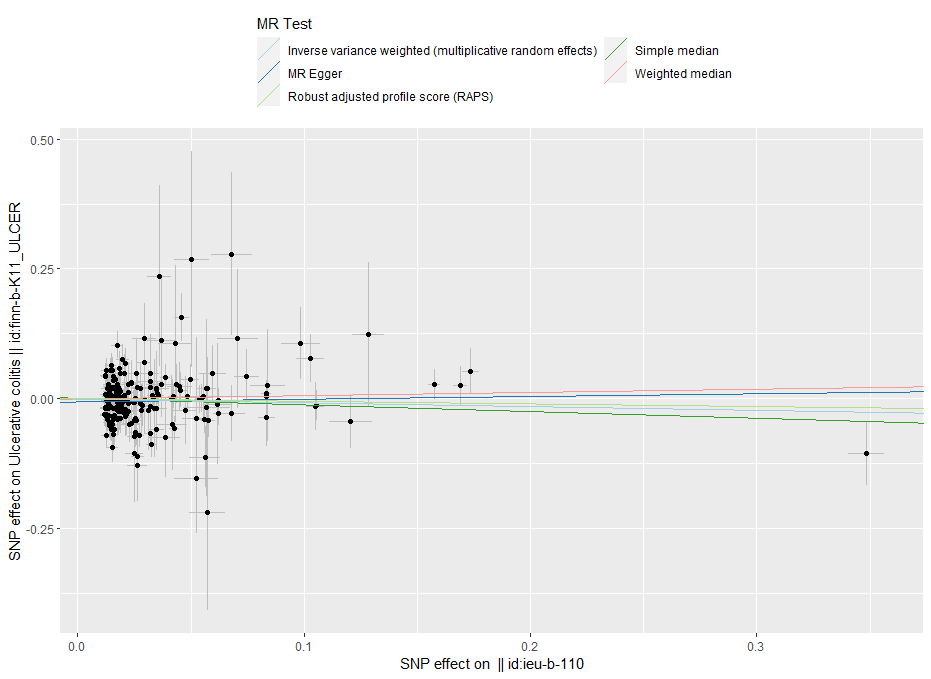


Supplemental Fig. 2C represent the effect of the trait on CD


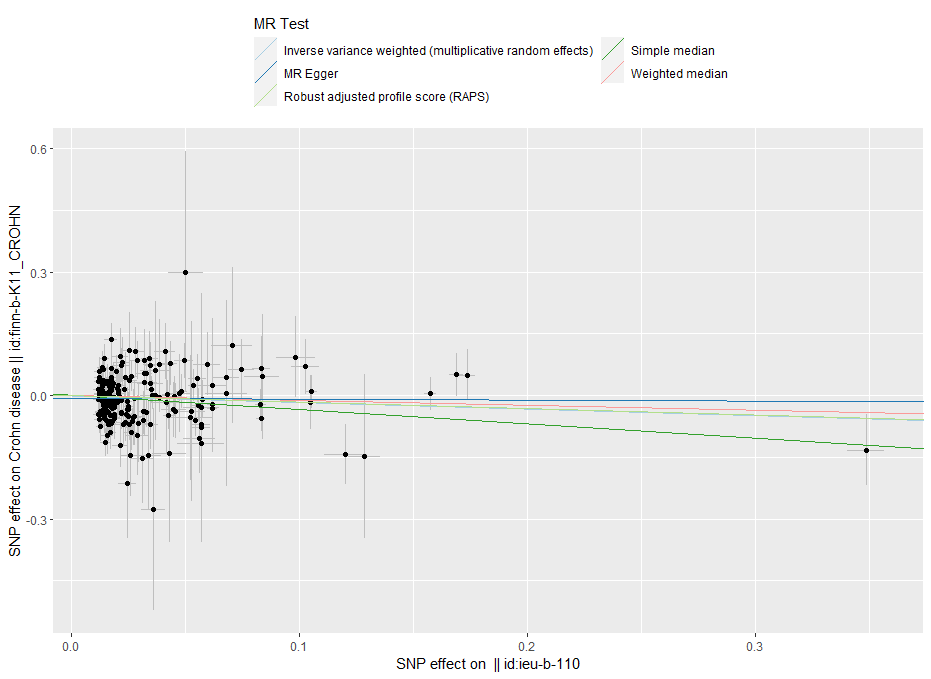


Supplemental Fig.3. Scatter plots showing the effect of TG on the outcome via different methods

(A), (B), (C) represent the effect of the trait on all IBD, UC and CD, respectively.

Supplemental Fig. 3A represent the effect of the trait on all IBD


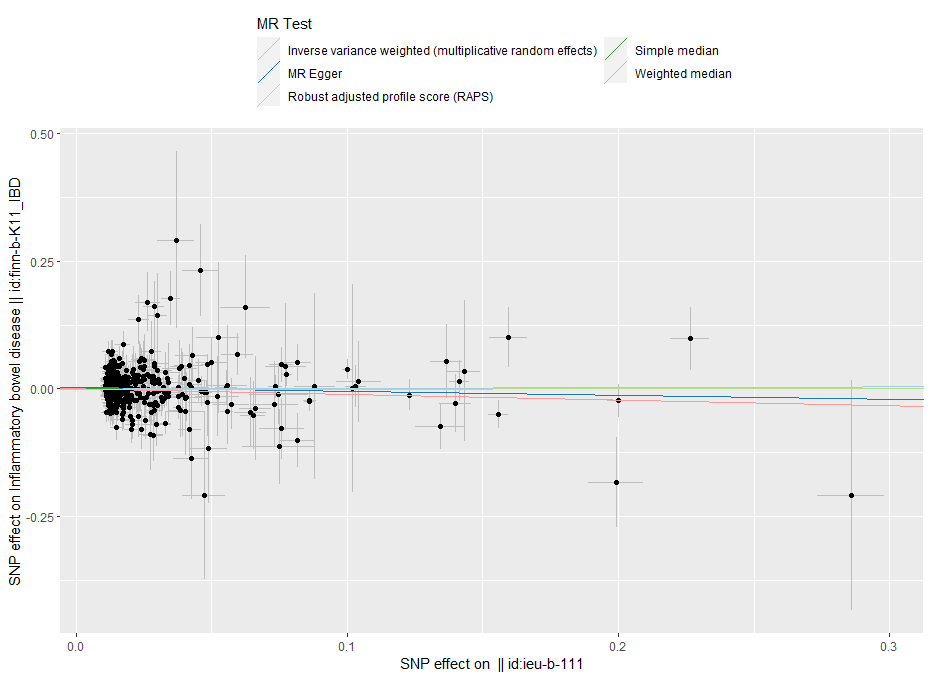


Supplemental Fig. 3B represent the effect of the trait on UC


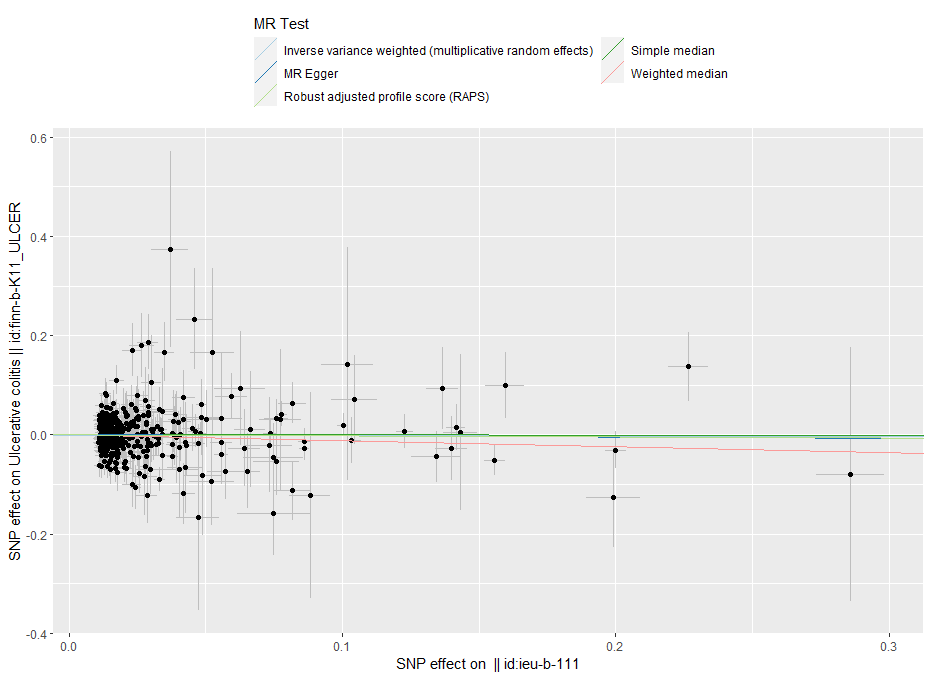


Supplemental Fig. 3C represent the effect of the trait on CD


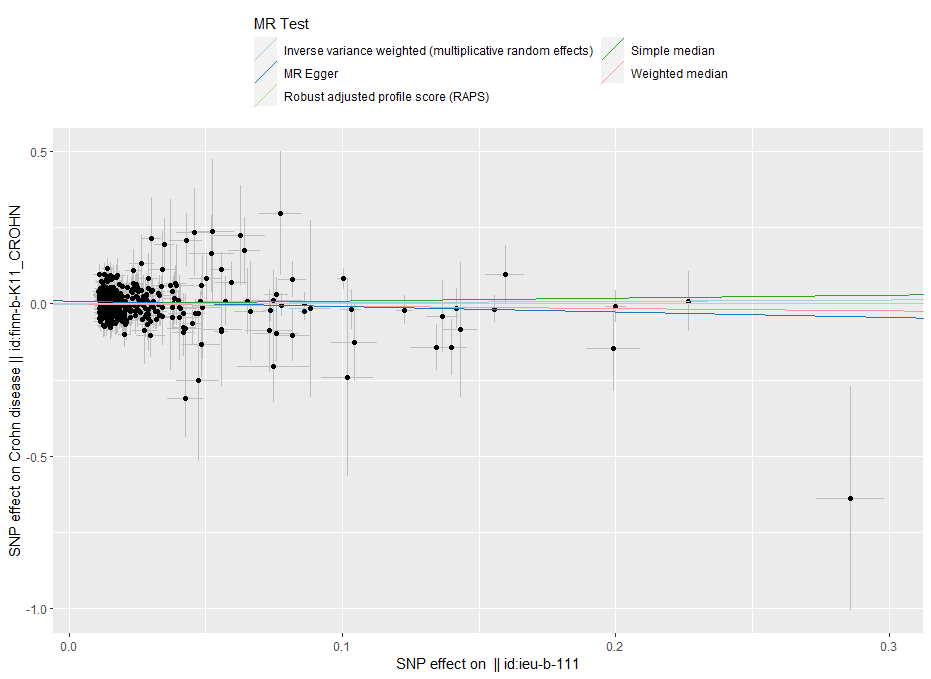


Supplemental Fig. 4. MR-Radial view of outliers of HDL-C for IBD, UC and CD

(A), (B), (C) represent the effect of the trait on all IBD, UC and CD, respectively.


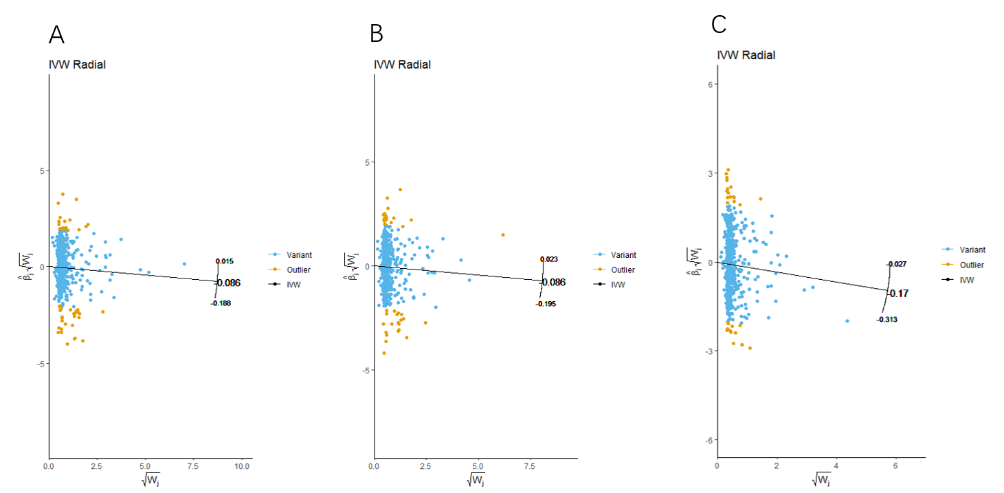


Supplemental Fig. 5. MR-Radial view of outliers of LDL-C for IBD, UC and CD

(A), (B), (C) represent the effect of the trait on all IBD, UC and CD, respectively.


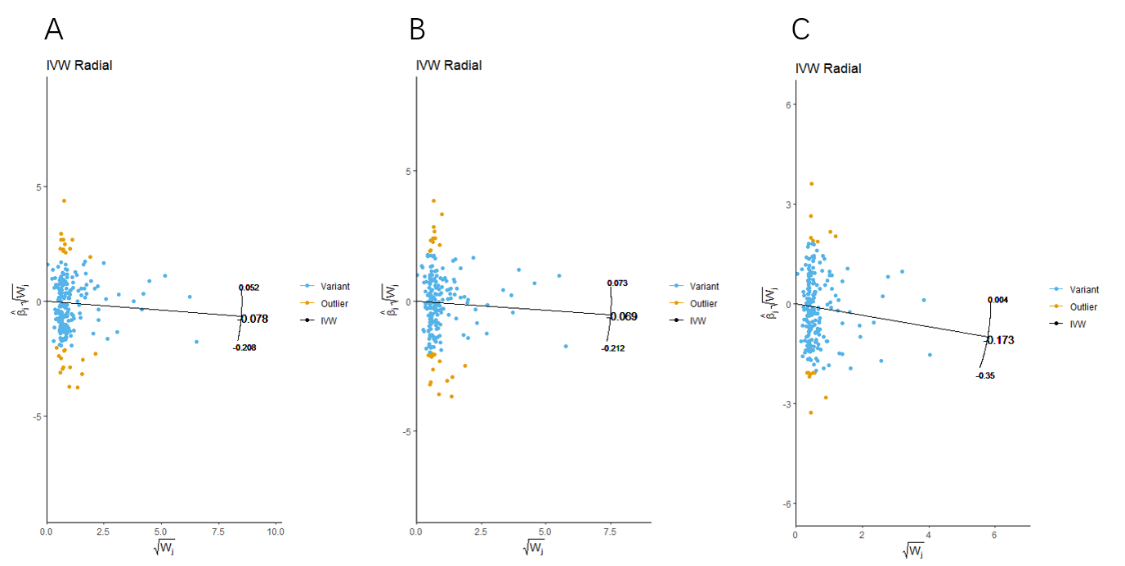


Supplemental Fig.6. MR-Radial view of outliers of TG for IBD, UC and CD

(A), (B), (C) represent the effect of the trait on all IBD, UC and CD, respectively.


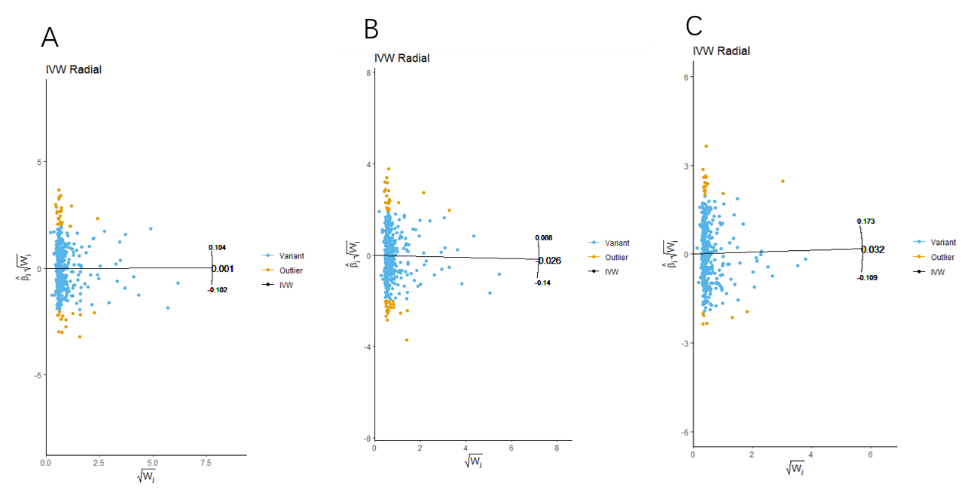


Supplemental Fig. 7. Robust regression and outlying variants penalized showed the trait on IBD, UC and CD, respectively.


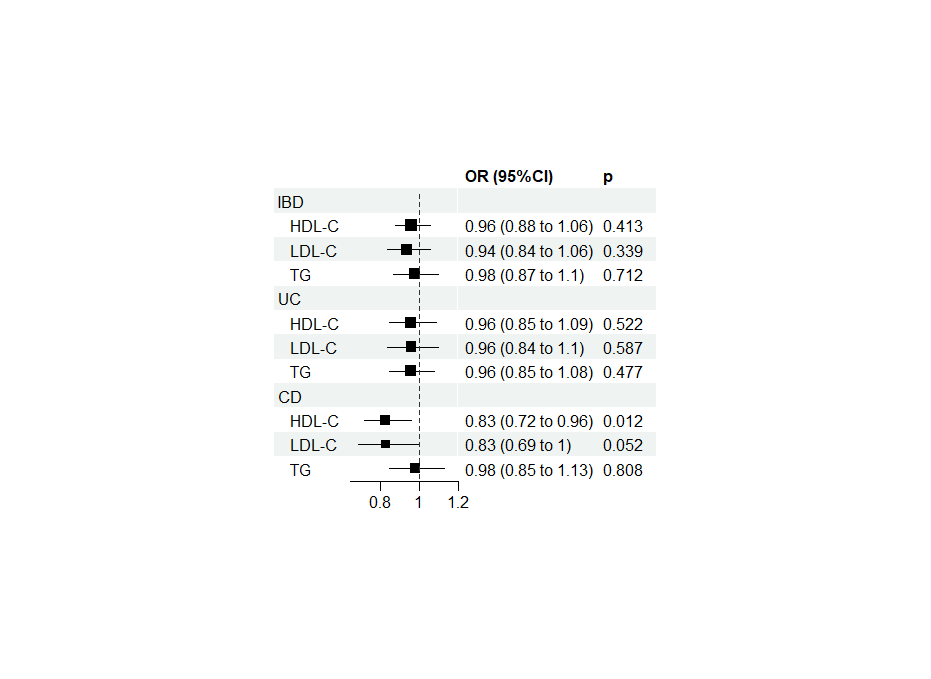


Supplemental Fig. 8. Leave-one-out analysis for the estimates of drug-target gene on IBD, UC and CD.

(A), (B), (C) represent the effect of the PSCK9 on IBD, UC and CD, respectively; (D) represent the effect of the CETP on CD.

Supplemental Fig. 8A. Leave-one-out analysis for the estimates of PSCK9 gene on IBD


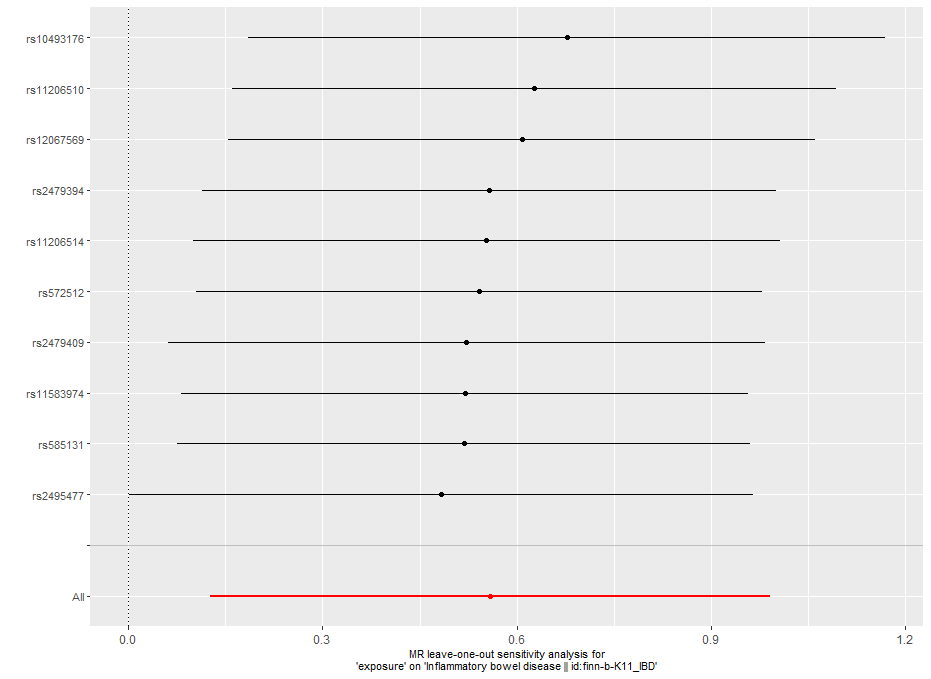


Supplemental Fig. 8B. Leave-one-out analysis for the estimates of PSCK9 gene on UC


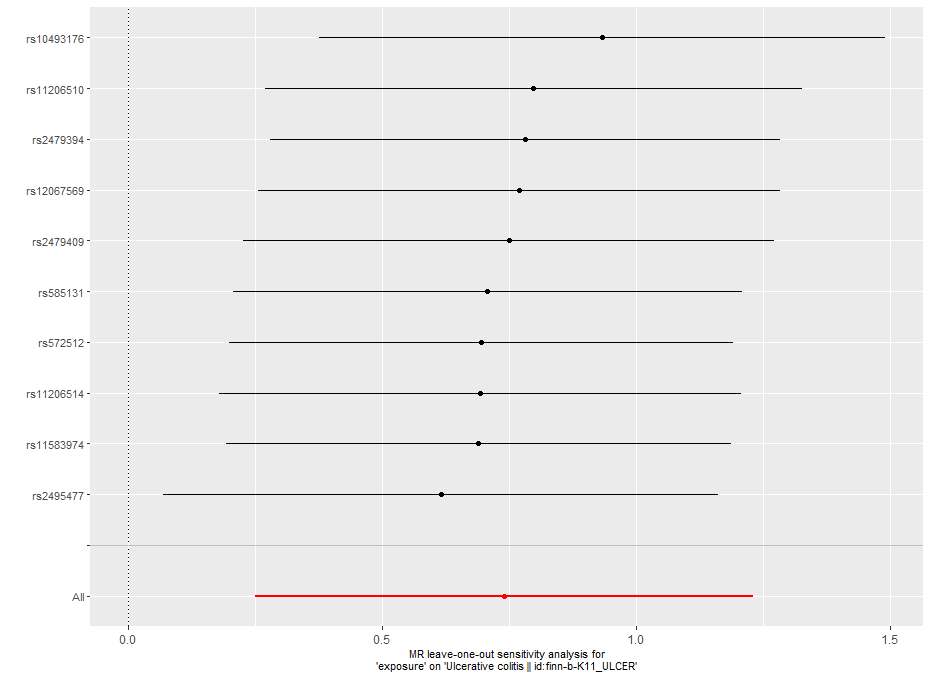


Supplemental Fig. 8C. Leave-one-out analysis for the estimates of PSCK9 gene on CD


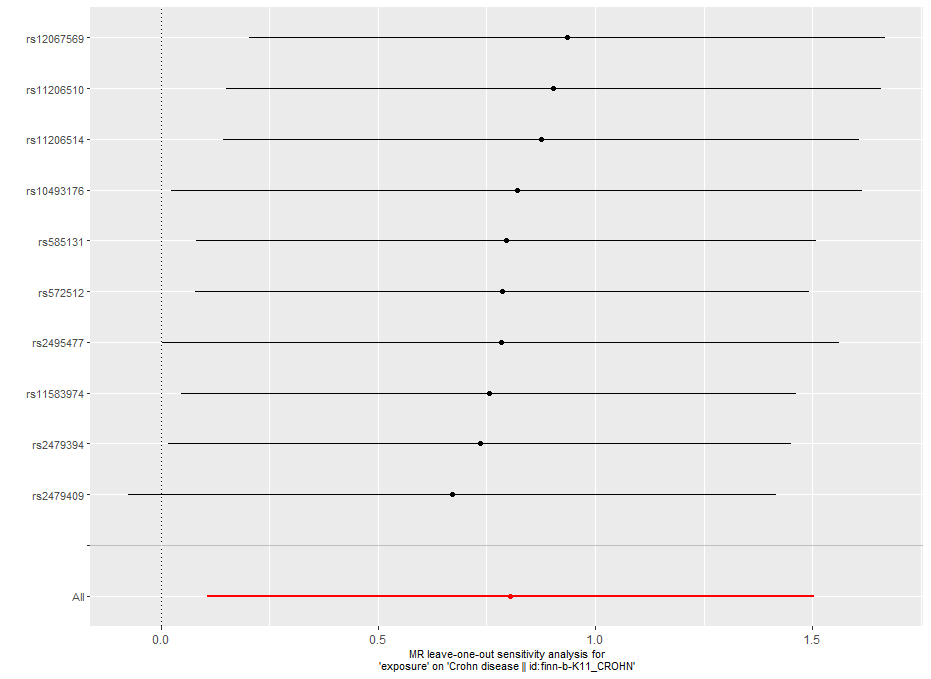


Supplemental Fig. 8D. Leave-one-out analysis for the estimates of CETP gene on CD


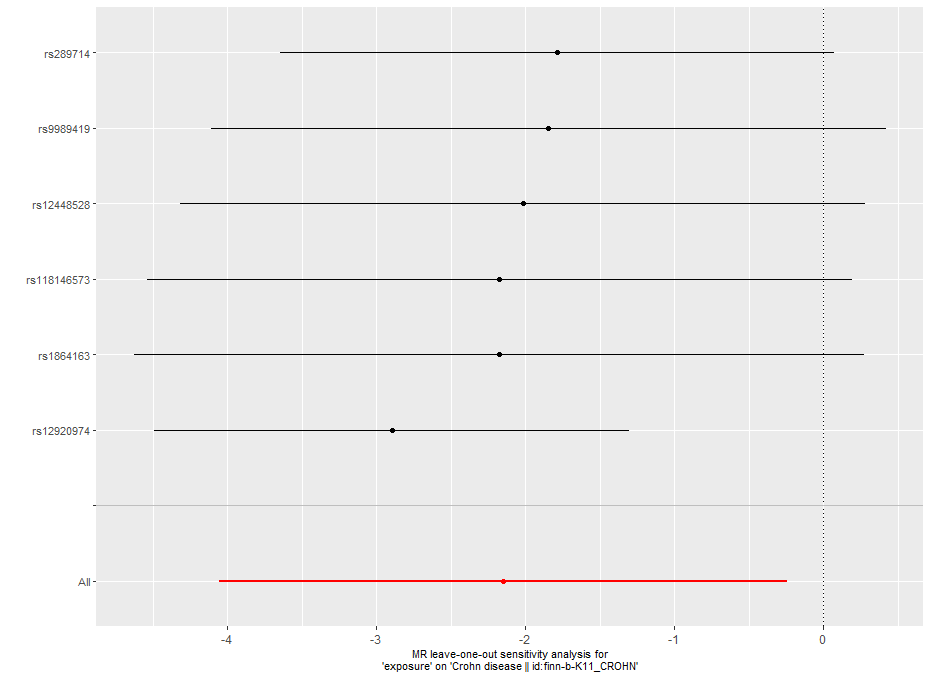


## Supplementary Tables

See the additional Excel file: Supplementary table.
